# Supplementary material for: Pathogen to commensal? Longitudinal within-host population dynamics, evolution, and adaptation during a chronic >16-year Burkholderia pseudomallei infection
Source: PLoS Pathog. 2020 Mar 5;16(3):e1008298. doi: 10.1371/journal.ppat.1008298 (PMC7077878; doi:10.1371/journal.ppat.1008298)
Supplement: S1 Fig — SNPs (17,359) from core genomic regions were identified and then used to construct a maximum parsimony tree of 11 ST131 isolates’ genetic relationships. K96243 was used as an outgroup for rooting. The two strains (MSHR1043 and MSHR6686) isolated from patient sputum clustered tightly with the six environmental isolates from the patient’s residence. The P314 reference genome is the closed genome of the environmental MSHR1435. The CI of this phylogenetic reconstruction was 0.96, which is indicative of a highly clonal mode of genetic inheritance. Over 16,000 SNPs separate the P314 cluster (clinical and associated environmental isolates) from the next nearest ST131 strains. (PDF) [file ppat.1008298.s001.pdf]

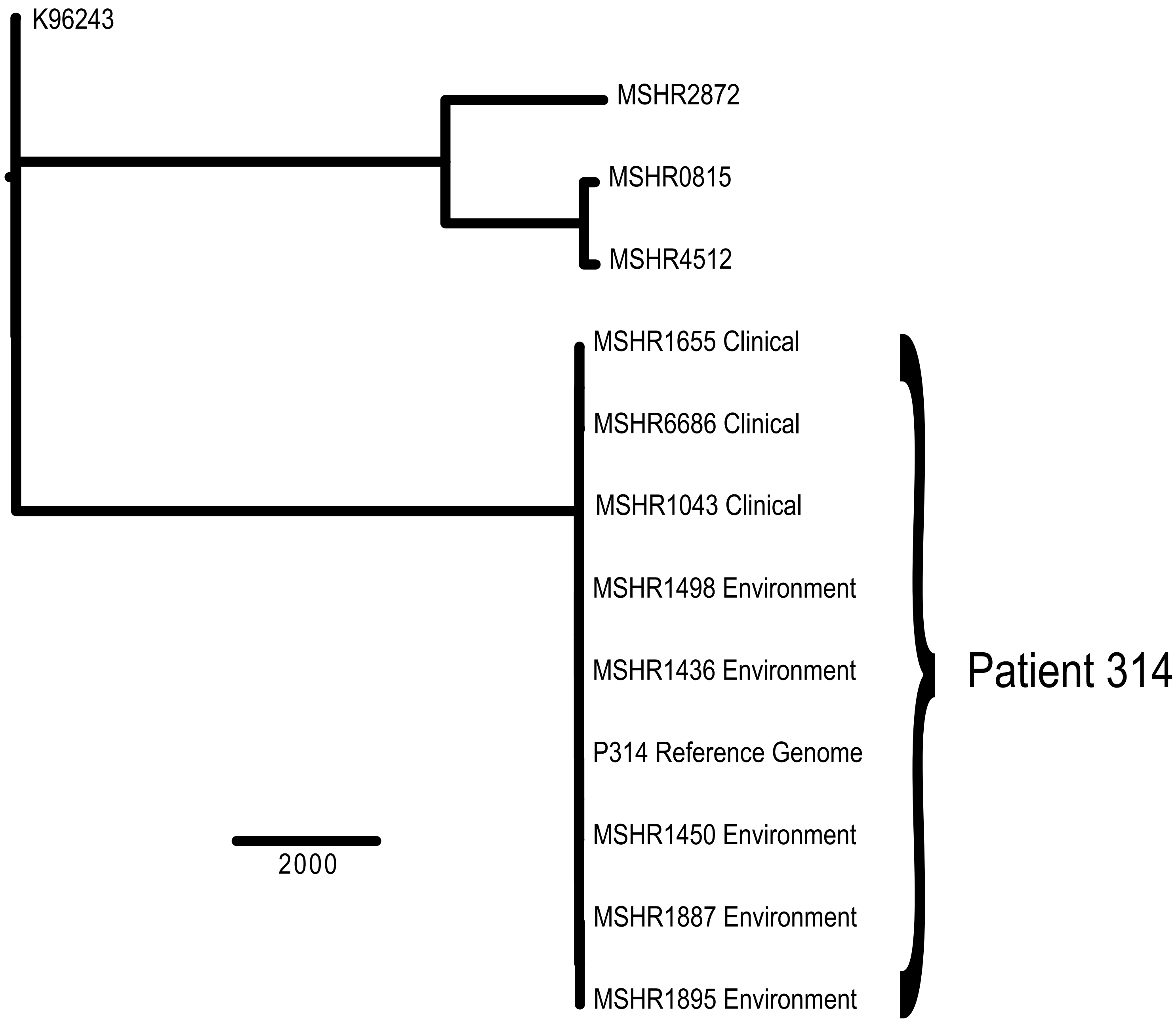

S1 Fig. Core genome relationships among ST131 isolates.
SNPs (17,359) from core genomic regions were identified and then used to construct a maximum parsimony tree of 11 ST131 isolates' genetic relationships. K96243 was used as an outgroup for rooting. The two strains (MSHR1043 and MSHR6686) isolated from patient sputum clustered tightly with the six environmental isolates from the patient's residence. The P314 reference genome is the closed genome of the environmental MSHR1435. The CI of this phylogenetic reconstruction was 0.96, which is indicative of a highly clonal mode of genetic inheritance. Over 16,000 SNPs separate the P314 cluster (clinical and associated environmental isolates) from the next nearest ST131 strains.
