# Supplementary material for: Pathogen to commensal? Longitudinal within-host population dynamics, evolution, and adaptation during a chronic >16-year Burkholderia pseudomallei infection
Source: PLoS Pathog. 2020 Mar 5;16(3):e1008298. doi: 10.1371/journal.ppat.1008298 (PMC7077878; doi:10.1371/journal.ppat.1008298)
Supplement: S2 Fig — Maximum parsimony tree from Fig 3 with branch labels. S2 Table lists all polymorphisms and includes information on the branch where the mutation occurred. (PDF) [file ppat.1008298.s002.pdf]

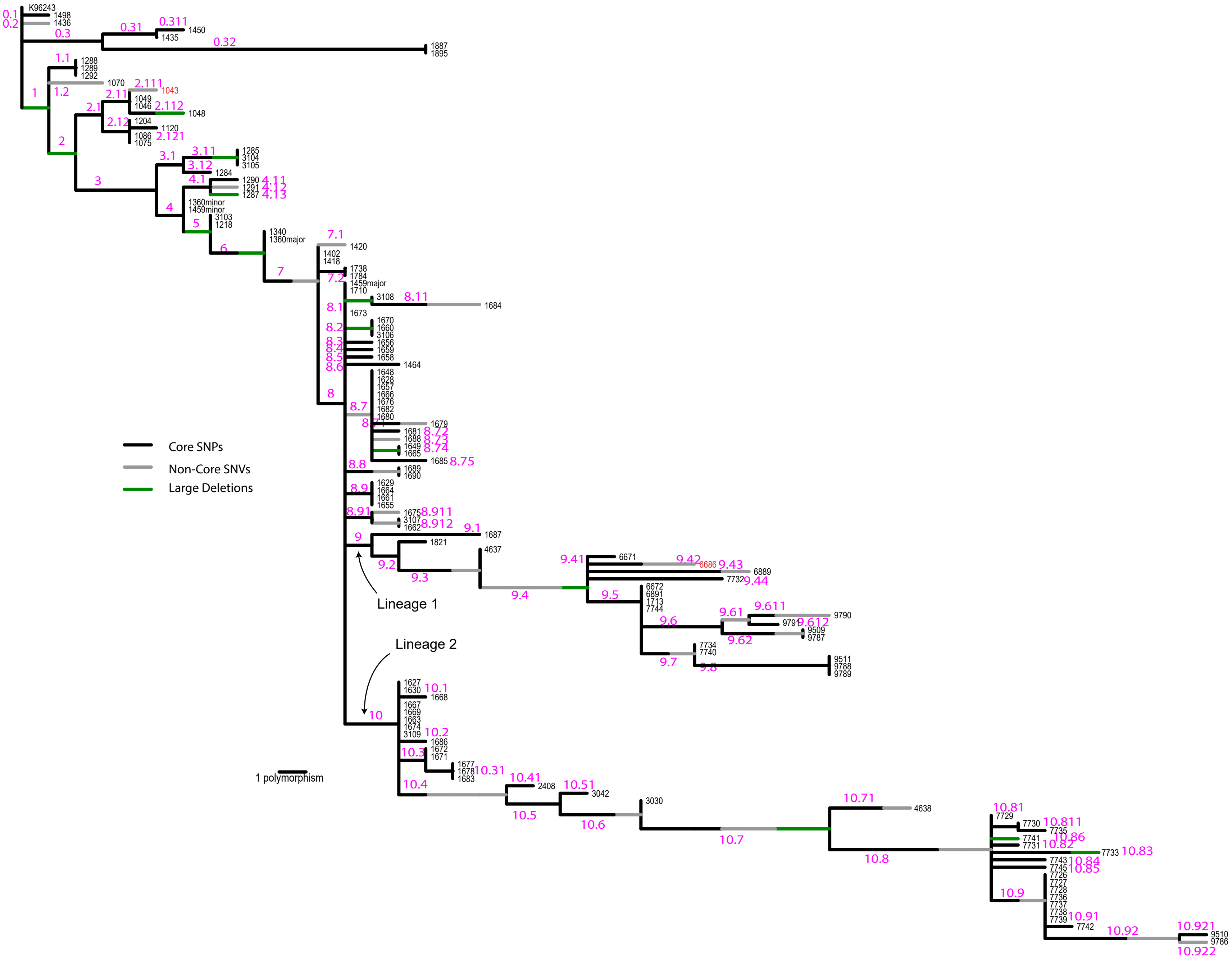


S2 Fig: Tree for branch labels. Maximum parsimony tree from Fig 3 with branch labels. Table 1 lists all polymorphisms and includes information on the branch where the mutation occurred.
