# Supplementary material for: Pathogen to commensal? Longitudinal within-host population dynamics, evolution, and adaptation during a chronic >16-year Burkholderia pseudomallei infection
Source: PLoS Pathog. 2020 Mar 5;16(3):e1008298. doi: 10.1371/journal.ppat.1008298 (PMC7077878; doi:10.1371/journal.ppat.1008298)
Supplement: S3 Fig — A-D) BALB/c mice were intranasally inoculated with B. pseudomallei isolates (n = 5/group). Mice were weighed and clinically scored daily. A) The average change ± standard deviation in weight of the group over time is shown for the initial 6 day period. B) The average clinical score ± standard deviation of the group over time is shown for the initial 6 day period. An increase in clinical score indicate an accumulation of clinical symptoms or an increase in morbidity. C) Individual clinical scores at day 2 are shown by dots (n = 5/group) and the mean score is shown by the bar. The isolates are listed by the relatedness to the environmental isolate cluster (right to left). (PDF) [file ppat.1008298.s003.pdf]

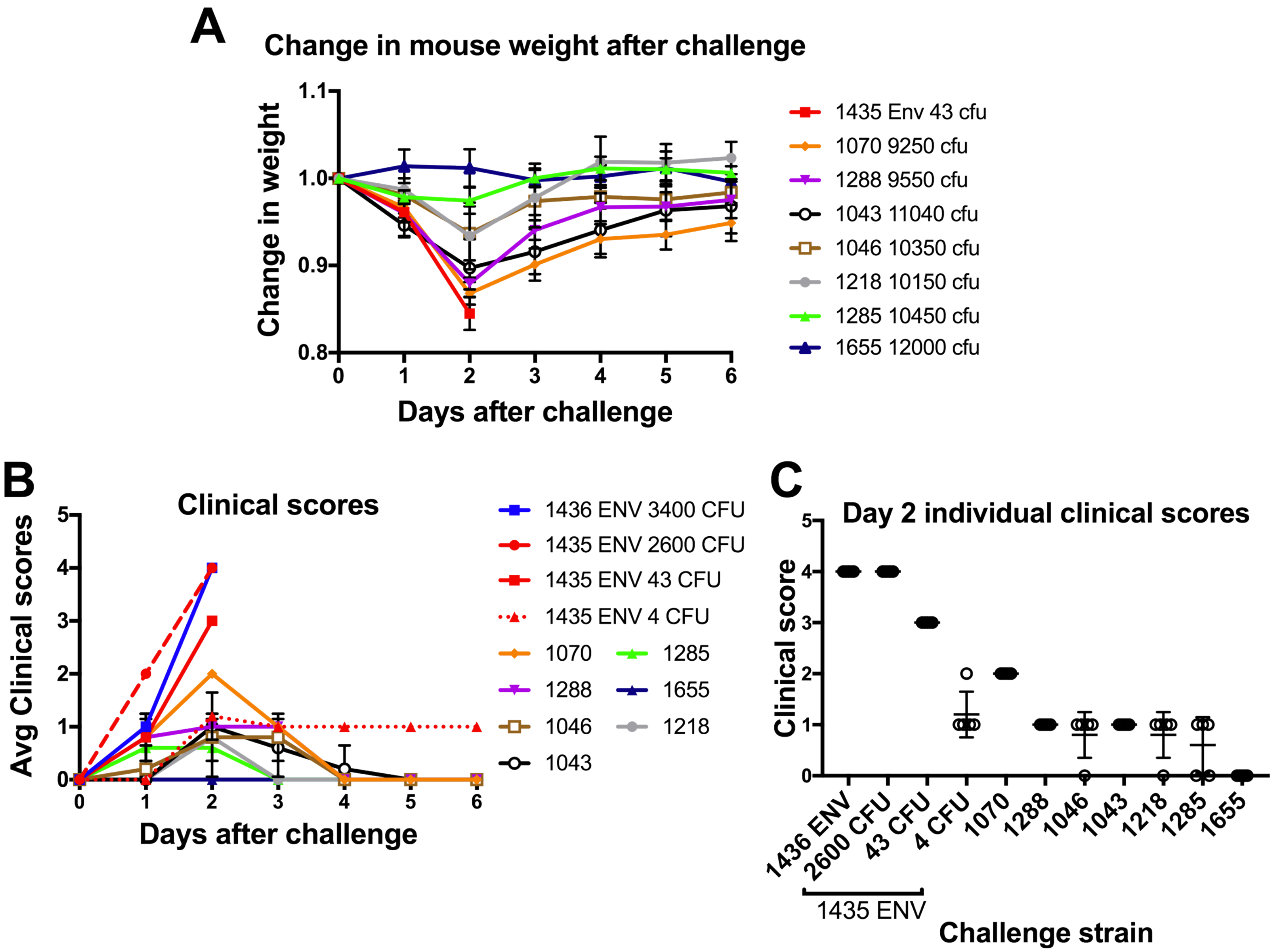

S3 Fig. Morbidity of P314 isolates and P314-associated environmental isolates. A-D) BALB/c mice were intranasally inoculated with B. pseudomallei isolates (n=5/group). Mice were weighed and clinically scored daily. A) The average change ± standard deviation in weight of the group over time is shown for the initial 6 day period. B) The average clinical score ± standard deviation of the group over time is shown for the initial 6 day period. An increase in clinical score indicate an accumulation of clinical symptoms or an increase in morbidity. C) Individual clinical scores at day 2 are shown by dots (n=5/group) and the mean score is shown by the bar. The isolates are listed by the relatedness to the environmental isolate cluster (right to left).
