# Supplementary material for: Pathogen to commensal? Longitudinal within-host population dynamics, evolution, and adaptation during a chronic >16-year Burkholderia pseudomallei infection
Source: PLoS Pathog. 2020 Mar 5;16(3):e1008298. doi: 10.1371/journal.ppat.1008298 (PMC7077878; doi:10.1371/journal.ppat.1008298)
Supplement: S4 Fig — Similar slopes for isolates phylogenetically located before the divergence of the two long-lived lineages and those of the long-lived lineage 2 suggests similar rates of evolution. A smaller slope for isolates from the long-lived lineage 1 suggests a lower evolutionary rate, however relatively large root-to-tip distances for the earliest long-lived lineage 1 isolates may indicate a temporary jump in evolutionary rate. Extrapolation of root to tip distances (excluding the long lived-lineage 1 isolates) suggests divergence from a common environmental isolate ~308 days before presenting at the hospital. This is a slightly longer estimate than provided by BEAST (Fig 9) which also utilizes invariant sites to estimate mutation rates. (PDF) [file ppat.1008298.s004.pdf]

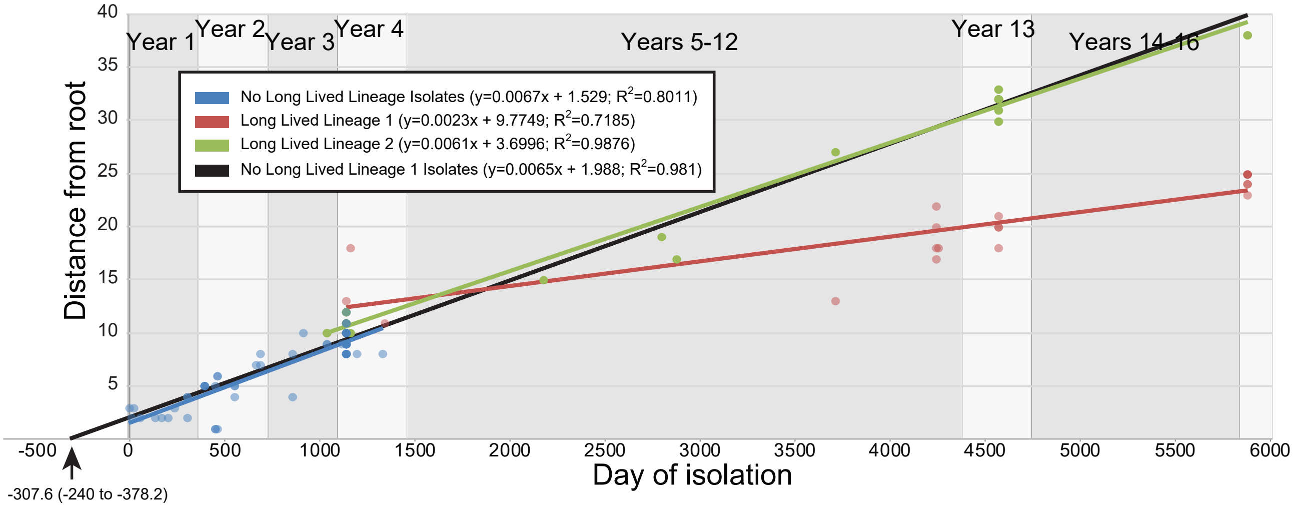

S4 Fig: Distances from the phylogenetic root to each genome. Similar slopes for isolates phylogenetically located before the divergence of the two long-lived lineages and those of the long-lived lineage 2 suggests similar rates of evolution. A smaller slope for isolates from the long-lived lineage 1 suggests a lower evolutionary rate, however relatively large root-to-tip distances for the earliest long-lived lineage 1 isolates may indicate a temporary jump in evolutionary rate. Extrapolation of root to tip distances (excluding the long lived-lineage 1 isolates) suggests divergence from a common environmental isolate ~308 days before presenting at the hospital. This is a slightly longer estimate than provided by BEAST (Fig 9) which also utilizes invariant sites to estimate mutation rates.
