# Supplementary figures and images for: Pathogen to commensal? Longitudinal within-host population dynamics, evolution, and adaptation during a chronic >16-year Burkholderia pseudomallei infection
Source: PLoS Pathog. 2020 Mar 5;16(3):e1008298. doi: 10.1371/journal.ppat.1008298 (PMC7077878; doi:10.1371/journal.ppat.1008298)

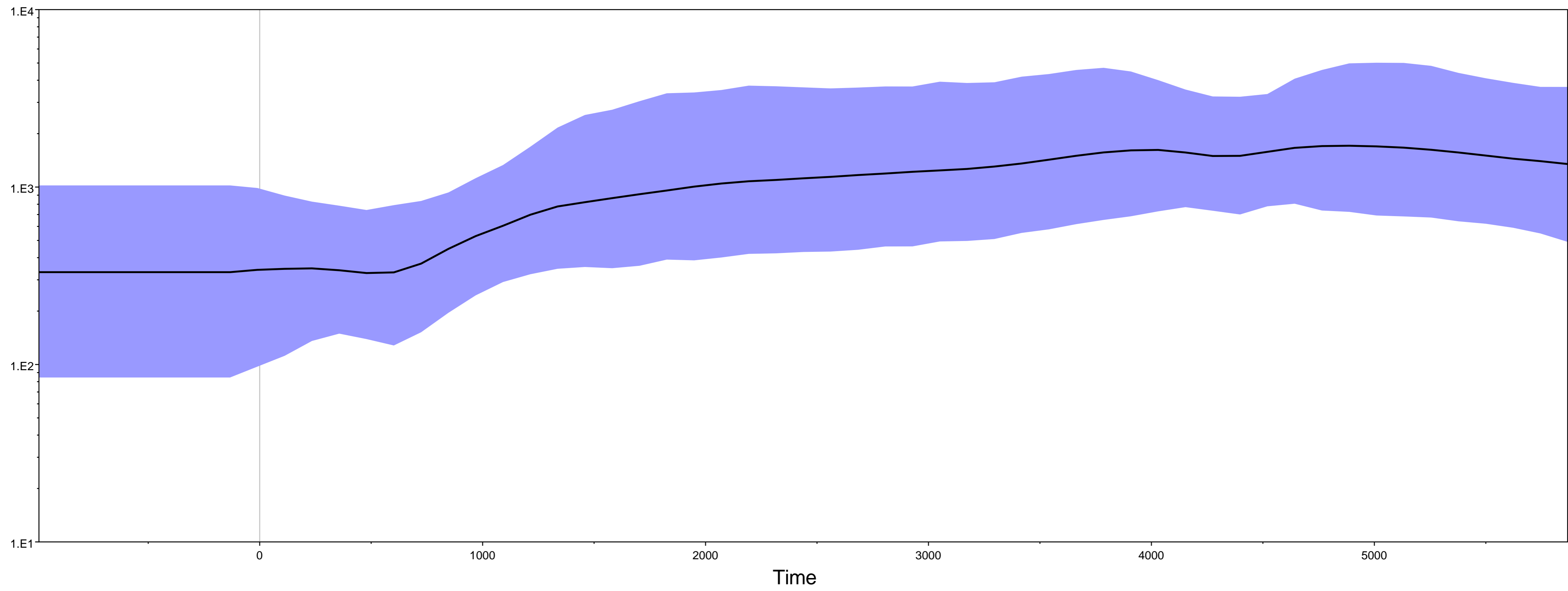

Supplement: S5 Fig — Downsampling of isolates collected at the time of the lobectomy show a similar estimation of effective population size compared to the full dataset. (PDF) [file ppat.1008298.s005.pdf]
